
JOURNAL INFORMATION
==============================
NLM Title Abbreviation: Atlanta Med Surg J (1884)
NLM Journal ID: 101251623

Atlanta Medical and Surgical Journal (1884)

Publisher: Biomedical Journal Digitization Project

ARTICLE INFORMATION
==============================
PMCID: PMC9201844
PMID: 35829601
Article version: 1
Subjects: Correspondence

Correspondence

Reeves C. S. M.D. Lone Grove, Texas

Print publication date: 1899 Jan
Volume: 15
Volume Series: New Ser
Issue: 11
First page: 743
Last page: 744
License: This work is free of known copyright restrictions. For more information, please see PMC Back Issue Digitization.
License URL: https://creativecommons.org/publicdomain/mark/1.0/

==============================

### CORRESPONDENCE.

Lone Grove, Texas, December 15, 1898.

*Dr. Dunbar Roy:*

Dear Sir.—I am just in receipt of the December number of your Journal, for which please accept thanks. I am a native Georgian; born equidistant between Monticello, in Jasper county, and Covington, in Newton county, 25th June, 1830. The "cold Saturday," in 1834, is about my first recollection. The "falling of the stars" in 1833 (November) was a little too early for me—only from hearsay. Have been a practitioner of the healing art since 1856—forty-two years. Your Dr. Tomlinson Fort, of Milledgeville, in Fort's Practice, gave me my first correct ideas of typhus and typhoid fevers. He placed it with other exanthemata and turned the attention of the profession of that era to the peculiar affection of the glands of the small intestines. What changes in the supposed etiology and treatment of diseases in general since that time! The Woodbridge treatment aborts it? cuts it short ( (?) not much), and because I cannot be *forced* to adopt this modern fad, "Tray, Blanche and Sweetheart" have all been on my track and have cast me into the fiery furnace of their hottest displeasure. However, in patience I have possessed my soul, as in the case of Galileo, who invented the telescope; I still stick to my belief, all the modern disciples of Æsculapius to the contrary notwithstanding. As surgeon in the war between the States I considered it fortunate that our medical armament consisted of spirits turpentine, blue mass, opium and quinine only. The hypodermic syringe and the various preparations of *coal-tar* were unknown. The turpentine emulsion, with *plenty of water* and proper diet, enabled me to help through the crisis of this disease scores of patients who would have died under the modern treatment; at least so I think.

There are several items in your JOURNAL I can heartily commend.

With your old physicians of thirty years ago I was once well acquainted. Ford, of Augusta; Hon. Dr. Miller, Fears, Shackleford, and many others, most of whom have joined the silent majority. Does my old friend and *chum*, Colonel Israel Putnam, still live? If so, remember me kindly to him when you see him. By the way, what think you of our proposed "Law to regulate the practice of medicine in Texas"? Too cumbersome, too wordy. One board of medical examiners to meet quarterly at the capitol would have been sufficient, I think. I doubt its passing the Legislature.

You are at liberty to use this letter or any part of it as you like. I write it in great bodily pain. I suppose that I have cancer of the rectum, growing out of hemorrhoidal tumors, from which I have suffered for near half a century.

Wishing you a pleasant Christmas, a happy new year, and success for your JOURNAL, I am,

C. S. REEVES, M.D.

[NOTE.—It gives us pleasure to publish this reminiscent letter of Dr. Reeves. We wish there were others of the "old school" who would do likewise and give the readers of THE JOURNAL some ideas from "ante-bellum days."]

---

### LOCAL APPLICATION IN GOUT.

℞ Potassii iodi<br>
Liniment saponis } aa .................................................. ʒ iv.<br>
Ol. cajaputi<br>
Ol. cari } aa .................................................. ʒ ss.<br>
Spiritus rect .................................................. q. s. ad. ℥ vii.<br>
M. Sig. Apply on lint and cover with a protective.

---

**NOTICE.—This JOURNAL and THE INTERNATIONAL JOURNAL OF SURGERY one year for $2.00 cash.**
